# Linkage to HIV Care Following HIV Self-testing Among Men: Systematic Review of Quantitative and Qualitative Studies from Six Countries in Sub-Saharan Africa

**DOI:** 10.1007/s10461-022-03800-8

**Published:** 2022-09-12

**Authors:** Mbuzeleni Hlongwa, Khumbulani Hlongwana, Sizwe Makhunga, Augustine T. Choko, Tafadzwa Dzinamarira, Donaldson Conserve, Alexander C. Tsai

**Affiliations:** 1 Discipline of Public Health, School of Nursing and Public Health, University of KwaZulu-Natal, Durban, South Africa; 2 Burden of Disease Research Unit, South African Medical Research Council, Cape Town, South Africa; 3 Public Health Group, Malawi Liverpool Wellcome Trust Clinical Research Programme, Chichiri, Blantyre, Malawi; 4 School of Health Systems and Public Health, University of Pretoria, Pretoria, South Africa; 5 Department of Prevention and Community Health, Milken Institute School of Public Health, The George Washington University, Washington, DC USA; 6 Center for Global Health and Mongan Institute, Massachusetts General Hospital, Boston, MA USA; 7 Harvard Medical School, Boston, MA USA; 8 Mbarara University of Science and Technology, Mbarara, Uganda

**Keywords:** HIV testing, HIV self-testing, Linkage to care, Men, Stigma, Sub-Saharan Africa

## Abstract

Gender disparities are pervasive throughout the HIV care continuum in sub-Saharan Africa, with men testing, receiving treatment, and achieving viral suppression at lower rates, and experiencing mortality at higher rates, compared with women. HIV self-testing (HIVST) has been shown to be highly acceptable among men in sub-Saharan Africa. However, evidence on linkage to HIV care following a reactive HIVST result is limited. In this systematic review, we aimed to synthesize the quantitative and qualitative literature from sub-Saharan Africa on men’s rates of linkage to HIV care after receiving a reactive HIVST result. We systematically searched 14 bibliometric databases. The Preferred Reporting Items for Systematic Reviews and Meta-Analysis (PRISMA) flow diagram was used to document the screening results. The Mixed Methods Appraisal Tool (MMAT) was used to assess the methodological quality of the included studies. Of 22,446 references screened, 15 articles were eligible for inclusion in this review. Linkage to HIV care following a reactive HIVST result was subject to several barriers: financial constraints due to travelling costs, potential long waiting hours at the clinics, stigma, discrimination, and privacy concerns. Men’s rates of seeking confirmatory testing and linking to HIV care following a reactive HIVST result were inconsistent across studies. Combining financial incentives with HIVST was found to increase the likelihood of linking to HIV care following a reactive HIVST result. The variable rates of linkage to HIV care following a reactive HIVST result suggest a need for further research and development into strategies to increase linkage to HIV care.

## Background

Gender disparities are pervasive throughout the HIV care continuum in sub-Saharan Africa. Compared with women, men test, receive treatment, and achieve viral suppression at lower rates; have higher mortality rates; and have greater loss to follow up after testing and treatment [1–7]. Studies conducted in multiple sites throughout sub-Saharan Africa have shown that men’s access to HIV-related services at healthcare clinics is adversely affected by multiple factors, including stigma, privacy and confidentiality concerns, long queues, financial barriers, and fear of testing positive [8–10]. As a result, men delay seeking HIV-related health care services and initiate HIV care and antiretroviral therapy (ART) at advanced stages of disease [11–15]. This phenomenon contributes to an increased HIV-related mortality rate among men within the first 3 months of starting ART, a rate that is nearly twice than that among women [13].

Early diagnosis of HIV infection is imperative for ensuring that people with HIV are started on treatment as soon as possible after diagnosis, with the aim of improving health outcomes, increasing life expectancy, and reducing secondary transmission. This objective lends urgency to calls to design strategies aimed at improving men’s uptake of HIV testing and progression throughout the HIV care continuum, so that the UNAIDS 95-95-95 goals can be achieved [16]. The last decade has witnessed various interventions being designed and implemented throughout sub-Saharan Africa that are aimed primarily at encouraging men to test for HIV, including community and home-based HIV testing and counselling, as well as antenatal clinic-based HIV testing for men attending with their partners [17, 18].

These HIV testing interventions have made some progress in improving HIV testing uptake among men in sub-Saharan Africa [17]. However, some HIV testing interventions, such as home based testing, may have limited community reach because men are less likely to be at home due to employment (and/or migratory employment) [17, 18]. Home based HIV testing includes mobile outreach and door-to-door testing and can be provided by lay people who have received certified HIV testing training [19]. National algorithms for testing and validating results should be followed where applicable [19].

The widespread acceptability of HIV self-testing (HIVST) has increased enthusiasm for deployment of this testing strategy to increase rates of HIV testing among men [17, 20, 21]. HIVST offers a new approach that may improve men’s HIV testing rates by removing some of the traditional barriers associated with accessing clinic-based HIV testing services and by enabling individuals to conduct and interpret their own HIV tests at their own convenient time and in their chosen private space [22–24]. HIVST can be based on either oral fluid or blood specimens. The HIVST user with a reactive test result is prompted to visit a healthcare clinic for confirmation of a reactive result and, if confirmed as HIV positive, link to HIV care. HIVST has been shown to be highly accurate, with sensitivity exceeding 90% and specificity exceeding 95%, suggesting a high probability of similar HIV results when confirmed at a healthcare clinic [25, 26]. Although HIV self-tests are generally reliable, varying rates of HIV in the population and/or user error might alter their sensitivity, specificity, and positive/negative predictive values [27]. User error has been identified as a leading cause of low sensitivity and specificity, with studies highlighting the roles of variables such as poor vision, insufficient lighting, and failure to read the results within the stipulated time period [28, 29].

Linkage to HIV care is defined as having obtained a confirmatory HIV test at a health care facility or designated community setting and, if confirmed as HIV positive, starting ART within 30 days [30]. However, measuring or monitoring linkage to HIV care following reactive HIVST results has been raised as an issue of concern in sub-Saharan Africa given the private nature of HIVST administration [31]. Further, optimum systems for linking HIVST clients to HIV care are not well established in sub-Saharan Africa [26].

There is limited information about linkage to HIV care following a reactive HIVST result, especially among men. The HIVST strategy has been implemented in phases, with the first phase involving evaluation of products and delivery models in three sub-Saharan African countries: Malawi, Zambia and Zimbabwe. The second phase of the HIVST strategy laid the groundwork for rapid scale-up and included three additional countries: South Africa, Lesotho, Eswatini. The third phase included further scale-up in five additional countries: Cameroon, Mozambique, Nigeria, Tanzania and Uganda [32]. By the end of 2020, over 4.8 million HIVST kits had been distributed in these countries [32]. However, the extent to which men in sub-Saharan Africa link to HIV care following a reactive HIVST result is not well characterized. While more research is being done around the world to develop a better understanding of HIVST effectiveness and to develop innovative techniques for targeting hard-to-reach persons, more research is needed in sub-Saharan Africa, especially given the high incidence of HIV [33]. Understanding the rates, as well as challenges and facilitators, of linkage to HIV care following a reactive HIVST result is critical to facilitating the scale-up of HIVST among men in sub-Saharan Africa. Collating and synthesizing these data may facilitate understanding of the structural and individual variables that obstruct linkage to HIV care after a reactive HIVST result and may also guide program implementaction, identify research needs, and influence policy development. To address this gap in the literature, we aimed to summarize the evidence about rates of linkage to HIV care following a reactive HIVST result, drawing from both quantitative and qualitative studies of men in sub-Saharan Africa.

## Methods

### Design

We undertook a systematic search for published and unpublished (grey) literature to synthesize evidence about the extent to which men in sub-Saharan Africa link to HIV care after a reactive HIVST result. Both qualitative and quantitative studies were eligible for inclusion if they described rates of linkage to HIV care among men after a reactive HIVST result, or men’s experiences with/perspectives about linkage to HIV care following a reactive HIVST result. This review followed the Preferred Reporting Items for Systematic Reviews and Meta-Analyses (PRISMA) guidelines [34] and the Population, Concept, and Context (PCC) framework for determining the eligibility of research questions (Table 1). Because this review was based on data reported in publicly available bibliometric databases and publications, it was exempted from ethical approval. This review did not involve human/animal participants; therefore, informed consent was not necessary.

### Identifying the Research Question

What is the available evidence on linkage to HIV care following a reactive HIVST result among men in sub-Saharan Africa?

### Suitability of the Question for a Systematic Review

See Table 1.

**Table 1:** Population, Concept, and Context framework

Criteria	Determinants
Population	Men aged 15 years and older in sub-Saharan Africa
Concept	Linkage to HIV care following a reactive HIVST result
Context	HIV epidemic in sub-Saharan Africa

### Search Strategy

Our comprehensive electronic search strategy was conducted to include studies published between January 2005 and February 2019, supplemented by a manual search conducted in January 2022 to include recently published eligible studies to cover the gap between 2019 and 2022. This manual search enabled us to include five additional studies in this review. The evidence search for this review was conducted concurrently with the evidence search for another review, published recently, which synthesised evidence from sub-Saharan Africa about men’s perspectives on HIVST [20, 21]. The current review builds on the previous study by focusing specifically on linkage to HIV care following a reactive HIVST result. HIVST is a relatively new intervention in sub-Saharan Africa. Therefore, studies published prior to 2005 were excluded given that they were unlikely to reflect pertinent contextual information about the HIVST model in sub-Saharan Africa. We searched the following electronic databases: PubMed/MEDLINE, American Doctoral Dissertations via EBSCOhost, the Union Catalogue of Theses and Dissertations (UCTD), SA ePublications via SABINET Online and WorldCat Dissertations, Theses via Online Computer Library Center (OCLC), the Education Resources Information Center (ERIC), the Cumulative Index to Nursing and Allied Health Literature (CINAHL), PsycINFO, Embase, Sociological Abstracts, Scopus, and Google Scholar. We also searched for reports issued by the South African Medical Research Council (MRC) and Human Sciences Research Council (HSRC), as well as those posted to the World Health Organization (WHO) and Joint United Nations Programme on HIV/AIDS (UNAIDS) websites. Additional potential studies were also sought by reviewing the reference lists of eligible articles. Boolean terms (AND, OR) and Medical Subject Heading (MeSH) terms formed part of our search strategy. The key search words used were; ‘HIV testing’, ‘HIV self-testing’, ‘HIV self-screening’, ‘linkage to care’, ‘men’, ‘male’, and ‘sub-Saharan Africa’. Names of individual countries in sub-Saharan Africa, and truncated terms such as ‘East Africa’ and ‘Southern Africa’, were also included in the search to ensure retrieval of articles indexed using country-specific names or regional terms. Potential articles from the database search were exported to Endnote version 7 for further assessment and screening [35].

### Study Selection and Inclusion Criteria

A single author (MH) conducted the initial database search, which included screening titles of articles and ensuring that the full text of potentially eligible studies were obtained for abstract screening. Two authors (MH and SM) then independently conducted abstract and full text screening, in duplicate, to identify studies that met the following inclusion criteria:

- Focused on HIVST and subsequent linkage to HIV care
- Conducted in sub-Saharan Africa
- Included only men, or included both men and women with sex-stratified analyses presented
- Published between January 2005 and January 2022
- Included individuals aged 15 years and older

There were no language restrictions. Studies published prior to 2005 and those conducted outside of sub-Saharan Africa were excluded. We excluded studies exclusively focused on men who have sex with men, given the potentially confounding influences that result from the unique challenges they (and other members of UNAIDS-defined “key population” groups—sex workers, transgender people, people who inject drugs, and people who are incarcerated) face in accessing HIV care and related services [36–40]. Any disagreements about inclusion or exclusion were resolved by consensus, in which both reviewers discussed the identified study and either agreed on a final result or had their disagreement resolved by a third reviewer.

### Quality Appraisal

Two authors critically appraised the methodological rigor of the included studies. All included articles were critically assessed using the Mixed Methods Appraisal Tool (MMAT), version 2018 [41, 42].

### Data Extraction

We used Google Forms to abstract data on study characteristics and relevance. The following characteristics were identified: (a) author(s) and date of publication, (b) aim(s) or research questions, (c) primary source data (e.g. quotes from individuals), (d) study population, (e) mean age of participants, (f) gender of participants, (g) percentage of women, (h) percentage of men, (i) geographic setting (rural/urban), (j) study design, (k) type of intervention and outcomes, (l) most relevant finding, (m) most significant finding, (n) study limitations and implications, and (o) interpretations and conclusions as stated by the authors.

### Data Synthesis

Using NVivo software (version 11, QSR International, Melbourne, Australia), we followed a thematic synthesis approach to synthesize data from the included studies. This approach followed three key stages: (i) coding the findings of the included studies line-by-line; (ii) organizing these free codes into related areas to construct descriptive themes; and (iii) developing analytical themes. All coding was discussed thoroughly among members of the research team to confirm that the key components of each included article were not overlooked. We extracted quantitative data on the percentages or rates of men who linked to HIV care following a reactive HIVST result, and compared linkage to care rates with those estimated in other studies included in this review.

## Results

**Fig. 1:**
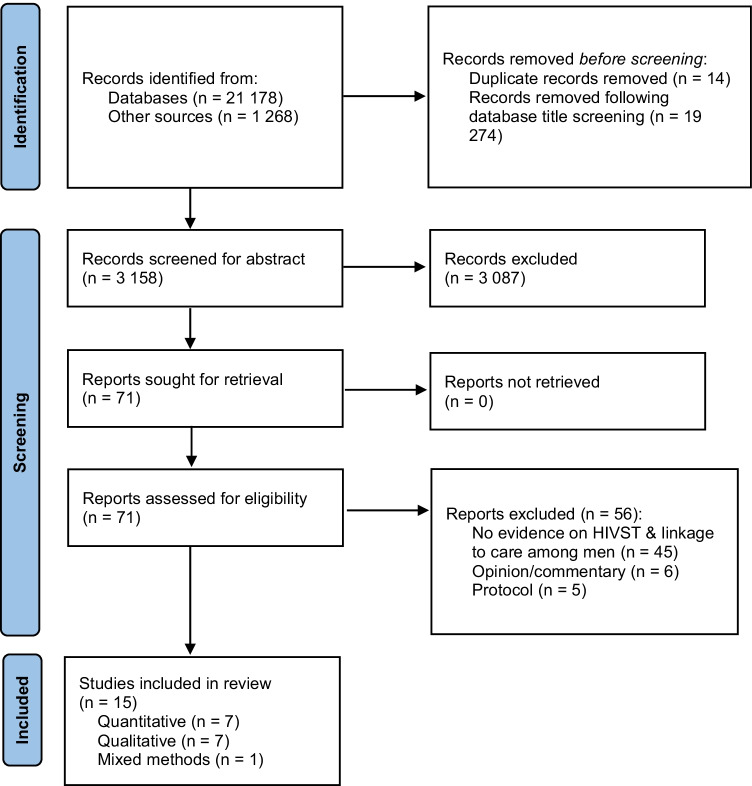
PRISMA flow diagram for systematic reviews

The electronic search strategy identified 22,446 references (Fig. 1). Title screening resulted in the exclusion of 19,274 articles. Fourteen duplicates were removed, leaving 3158 articles for title and abstract screening, resulting in the further exclusion of 3087 articles. We assessed the full text of 71 articles and excluded 56 of them for the following reasons: six were opinion/commentary papers, five were study protocols, and 45 did not present evidence on HIVST and linkage to HIV care among men in sub-Saharan Africa. Therefore, 15 articles met our inclusion criteria and were included in this review.

### Characteristics of Included Studies

The included studies were conducted in the following countries: Zambia [43], Malawi [26, 44, 45], Tanzania [46], Uganda [47–50], South Africa [51–54], and Kenya [55, 56]. There were seven quantitative studies, seven qualitative studies, and one mixed methods study. This review included studies published from January 2005 to January 2022. Our search excluded studies conducted prior to 2005 because they were unlikely to reflect key aspects and information pertaining to the use of the HIVST model in sub-Saharan Africa. All of the included studies were published after 2015. Details of the included studies are presented in Table 2, with the quality assessment presented in Appendix Table 3.

**Table 2:** Characteristics of included studies

Author (year)	Country	Study aim	Sample/population	Number of participants	Age group	Research method	Key finding(s)
*Qualitative studies*							
Muwanguzi et al. (2021)	Uganda	To explore employed professional men’s preferences for uptake of HIVST and linkage to HIV care or prevention services	Men	33	18–55 years	Qualitative (in-depth interviews)	Incentives could be used to improve the rates at which men are linked to HIV care following a reactive HIVST result
Rujumba et al. (2021)	Uganda	To explore perceptions of pregnant and lactating women, their male partners and health care providers regarding both initial and repeat HIVST for women and their male partners during pregnancy and lactation in Kampala and generated suggestions for potential integration and scale-up of HIV self-testing in PMTCT programs	Men & women	22 women 12 men 23 health care providers	24–40 years	Qualitative (in-depth interviews & focus group discussions)	Concern that, in addition to confidentiality concerns and stigma, the lack of active linkage to care systems could be another barrier to timely linkage
Matovu et al. (2020)	Uganda	To generate data necessary to inform the design of a peer-led HIVST intervention intended to improve HIV testing uptake and linkage to HIV care in Kasensero fishing community in rural Uganda	Men & women	16 women 31 men	15 years & older	Qualitative (focus group discussions)	Men preferred a home visit from a health care provider as a follow up strategy to encourage them to confirm reactive HIVST results and link to HIV care
Conserve et al. (2018)	Tanzania	To assess men’s attitudes and personal agency towards HIVST and confirmatory HIV testing in order to inform the development of the Tanzania Self-Testing Education and Promotion Project, a peer-based HIV self-testing intervention for young men in Tanzania	Men	23	Mean age: 27.3 years	Qualitative (in-depth interviews)	Men preferred phone call reminders over SMS reminders after HIVST
Choko et al. (2017)	Malawi	To describe the views of pregnant women and their male partners on HIV self-test kits that are woman-delivered, alone or with an additional intervention	Men & women	31 women 31 men	Median age for men: 28.5 years; women: 23.5 years	Qualitative (in-depth interviews & focus group discussions)	Men felt that providing a fixed financial incentive of approximately USD $2 would increase linkage to HIV care following a reactive HIVST result
Martinez Perez et al. (2016)	South Africa	To examine the feasibility and acceptability of unsupervised oral self-testing for home use in an informal settlement of South Africa	Men & women	11 women 9 men	18 years & older	Qualitative (couple interviews, in-depth interviews, focus group discussions)	Healthcare providers’ home visits may deter future utilization of HIVST. Concern that home visits could potentially stigmatize HIVST clients who are labelled as HIV infected
Makusha et al. (2015)	South Africa	To explore interest in HIVST; potential distribution channels for HIV self-test kits to target groups; perception of requirements for diagnostic technologies that would be most amenable to HIVST and opinions on barriers and opportunities for HIV-linkage to care after receiving positive test results	Men & women	2: Government Officials; 4: NGOs; 2: Donors; 3 Academic Researchers; 1 Int. stakeholder	18 years & older	Qualitative (in-depth interviews)	Some of the barriers to linkage to HIV care after a reactive HIVST result pertain to the lack of a personal referral system
*Quantitative studies*							
Sithole et al. (2021)	South Africa	To investigate whether HIV self-test kit distribution was a feasible approach to reach men and to estimate the proportion of participants who reported their HIVST results, tested positive, and linked to care	Men	2634	Median age: 27 years (interquartile range: 22–33)	Quantitative	65% (n = 102/157) and 70% (n = 110/157) of men were linked to ART after a reactive HIVST result. Men who received an HIVST kit at a place other than the workplace or mobile van (adjusted odds ratio [AOR] 3.58; 95% confidence interval [CI] 1.30–14.84; p = 0.033) and those with a secondary level of education or above (AOR 1.34; 95% CI 1.00–1.78; p = 0.046) were more likely to report their HIVST results
Shapiro et al. (2020)	South Africa	To understand whether HIV self-test kit distribution is feasible to engage men in testing, to determine the yield of HIV detection and linkage to care for men by providing HIVST in South African communities, and to determine predictors of retention along the HIV cascade for men who use HIVST, in order to better optimize engagement for men	Males & females	4307 men 189 women	18 years & older	Quantitative (implementation)	72% of men with a reactive HIVST result received a confirmatory test, with 95% of these linking to ART. Overall linkage was confirmed for 68% of HIV diagnosed men
Korte et al. (2020)	Uganda	To evaluate the impact of offering HIVST to male partners of women presenting for antenatal care (ANC)	Male partners of pregnant women attending ANC	1455	18 years & older (mean age [standard deviation] 32.2 [8.1] years)	Quantitative (cluster-randomized controlled trial)	23% (n = 6/26) of men in the intervention vs 66.7% (n = 4/6) in the control arm were linked to HIV care following a reactive HIVST result
Choko et al. (2019)	Malawi	To investigate the impact of HIVST alone or with additional interventions on the uptake of testing and linkage to care or prevention among male partners of ANC clinic attendees in a novel adaptive trial	Male partners of pregnant women attending ANC	2349	Mean age: 30 years	Quantitative (cluster-randomized controlled trial)	91.3% (n = 42/46) of men were linked to ART following a reactive HIVST result
Thirumurthy et al. (2016)	Kenya	To assess an approach of providing multiple self-test kits to women at high risk of HIV acquisition to promote partner HIV testing and to facilitate safer sexual decision making	Male partners of sex workers and women receiving antenatal and post-partum care	280	18–39 years	Quantitative (cohort study)	56% (n = 23/41) of men were linked to HIV care following a reactive HIVST result
Masters et al. (2016)	Kenya	To determine whether providing multiple HIV self-test kits to pregnant and postpartum women for secondary distribution is more effective at promoting partner testing and couples testing than conventional strategies based on invitations to clinic-based testing	Male partners of pregnant women attending ANC	570	18 years & older	Quantitative (randomized controlled trial)	25% (n = 2/8) were linked to ART following a reactive HIVST result
Choko et al. (2015)	Malawi	To evaluate uptake, accuracy, linkage to care, and health outcomes when highly convenient and flexible but supported access to HIV self-test kits were provided to a well-defined and closely monitored population	Men & women	7868 women 6124 men	16 years & older	Quantitative (prospective—within a cluster-randomised trial)	Linkage to HIV care after a reactive HIVST result was 56.3% (n = 524/930)
*Mixed*-*methods studies*							
Chipungu et al. (2017)	Zambia	To examine the intention to link to care amongst potential HIVST users and the suitability of three linkage to care strategies in Lusaka Province, Zambia	Men & women	Quantitative: 1617 (60% women, 40% men) Qualitative: 64 participants	16–49 years	Mixed methods: Quantitative (cross sectional) & qualitative (focus group discussions)	82% (n = 533/647) of men were willing to link to ART within the 1st week after a reactive HIVST result

### Key Themes

We identified three main themes: evidence on linkage to HIV care following a reactive HIVST result; barriers to linking to HIV care following a reactive HIVST result; and strategies to increase linkage to care following a reactive HIVST result.

### Evidence on Linkage to HIV Care Following a Reactive HIVST Result

Evidence on linkage to HIV care following a reactive HIVST result was presented in 12 studies (seven quantitative, four qualitative, and one mixed methods). In Zambia, 82% (n = 533/647) of men reported willingness to link to care within the 1st week after a reactive HIVST result [43]. A recent study of 3486 men conducted in South Africa showed that as high as 72% of men with a reactive HIVST result received a confirmatory test, and nearly all (95%) who were confirmed to be HIV positive were subsequently linked to HIV care and initiated ART [53]; overall, 68% of men with a reactive HIVST result were linked to confirmatory testing and initiated ART successfully. In a study conducted in KwaZulu-Natal, South Africa, linkage to HIV care was confirmed for 65% (n = 102/157) within 7 months of testing and 70% (n = 110/157) within 15 months of testing, among men who reported their results [54].

In Malawi, studies of secondary distribution of HIVST kits to male partners by women attending antenatal care found evidence of favorable opinions toward linkage to HIV care after a reactive HIVST result [44], especially when conditional financial incentives were included [45]. In another Malawi study, among 676 male partners of antenatal care clinic attendees who attended a male-friendly clinic (i.e., a clinic designed specifically for men and/or with male staff), 46 (6.8%) had a newly confirmed reactive HIVST result, all of whom were referred for ART; of these, 42 (91.3%) were linked to HIV treatment on the same day as their HIV diagnosis [45].

In the Zambia study, linkage to HIV care was lower among men receiving HIV testing for the first time compared with men who had prior HIV testing experience (adjusted odds ratio [AOR] 0.54; 95% confidence interval [CI] 0.32–0.91; p = 0.02), and was also lower among men with high incomes compared with men who had lower incomes (AOR 0.59; 95% CI 0.40–0.88; p = 0.009) [43]. While linkage to HIV care after a reactive HIVST result has been high in some studies, in a study conducted in Malawi, the authors estimated a much lower rate of linkage to HIV care after a reactive HIVST result (56.3%, n = 524/930) [26]. In Kenya, 63% (n = 26/41) of participants with a reactive HIVST result sought confirmatory testing, and 56% (n = 23/41) of those were linked to HIV care within 3 months [56]. Lower rates of linkage to HIV care after a reactive HIVST result were reported in Kenya, where only 25% (2/8) of men with a reactive HIVST result obtained confirmatory testing and subsequently linked to HIV care [55]. Similarly, only 23% (n = 6/26) of men who were HIVST reactive in Uganda were linked to HIV care in the intervention arm compared with 66.7% (n = 4/6) in the control arm [47].

In Zambia, the participants demonstrated an understanding that getting a confirmatory HIV test, seeking advice, and starting ART was indicated after a reactive HIVST result, especially because some people may require more support and counselling, as echoed by a Zambian man:

If a person is positive they need to find people who can help [them], so that they can be comforted and not have the feeling of saying ‘why have I been found positive or what can I do ([43], p. 7).

A study in Tanzania found that men’s positive attitudes towards seeking confirmatory HIV testing and linkage to HIV care were motivated by the availability of lifesaving treatment [46]:

Truly if I see two lines [reactive HIVST results] have appeared I will be ready to move from this place to the responsible place to verify my results. ([46], p. 8).

In another study conducted among male partners of antenatal care clinic attendees, men felt that providing a fixed financial incentive of approximately USD $2 would increase linkage to HIV care following a reactive HIVST result [44], citing the possibility that such a strategy would be used for transport costs, removing a crucial economic barrier:

When you come to the clinic, you spend the whole day with no food for today. Providing a high financial incentive would encourage other male partners, upon hearing that their friend just got food for the day by simply going to the clinic. ([44], p. 6).

However, also important to note is that these men would still have to deal with issues preventing them from attending an HIV clinic, including stigma and confidentiality concerns, long waiting times, lack of paid leave to attend clinic, and limited clinic operating hours [46, 53].

Some of the barriers to linkage to HIV care after a reactive HIVST result pertain to the lack of a personal referral system for linking people to HIV care [51]. Setting up an efficient monitoring and evaluation programme for HIVST is important, as well as the provision for adequate follow up after a reactive HIVST result [51, 52].

### Barriers to Linkage to HIV Care Following a Reactive HIVST Result

Evidence on the barriers associated with linkage to HIV care following a reactive HIVST result was presented in six studies. These consisted of three quantitative studies, two qualitative studies and one mixed methods study. Studies conducted in Zambia, Uganda, Malawi and South Africa indicated that men of limited financial means may not obtain confirmatory HIV testing and/or initiate ART due to financial constraints or travelling costs [43, 45, 47]. In a study conducted in Uganda, participants suggested that incentives could be used to improve the rates at which men are linked to HIV care following a reactive HIVST result:

Why would I want to take an HIV test? There must be a reason why, especially if you want me to take it at the office. But if you put a test and said those who take the test and go for treatment will be given something, then I would be willing to participate. ([50], p. 8).

You could even motivate them that you will give them a reward when they bring back the results or when they go for other services. For example, circumcision or when they start on treatment if they test positive. ([50], p. 9).

Choko et al. [26, 45] also indicated that, while there is high readiness to engage in HIVST, optimal systems for linking clients to HIV care are not well established in Africa.

Additional barriers to linkage to HIV care include the potentially long wait times at the clinics, stigma, discrimination, and privacy concerns [46, 47]. In a study conducted in Kampala, Uganda, some men expressed concern that, in addition to confidentiality concerns and stigma, the lack of active linkage to care systems could be another barrier to timely linkage:

In case he tested himself he will not encourage himself to go to the clinic and start taking ARVs. Some people will not even know which hospital to go to for treatment. Some fear going to hospitals near their homes because they do not want to be known ([48], p. 11).

### Strategies to Increase Linkage to Care Following a Reactive HIVST Result

Evidence about the most appropriate strategies for increasing follow up after HIVST was reported in eight studies [43–46, 49, 50, 52, 54]. These consisted of five qualitative studies, two quantitative studies, and one mixed methods study. A study conducted in Zambia indicated that more than half (51%; n = 328/647) of men preferred a home visit from a health care provider as a follow up strategy for confirming reactive HIVST results and linking to HIV care, citing the enhanced privacy, greater trust, and more effective communication they perceived with home visits compared with health facilities [43]. This finding was consistent with the findings from a study conducted in Rakai, Uganda, although study participants also noted concerns over potential breaches of confidentiality that could result from home visits:

In my opinion it’s a good [idea] because no transport cost is involved by self-tested individuals. However, if community members become aware that a medical worker always visits a certain person in the village, they become suspicious that the person is HIV positive which may make the one [who] obtains ARVs from his home uncomfortable ([49], p. 11).

In another study conducted in Uganda during the COVID-19 pandemic, men indicated that follow up telephone calls from health care providers would be preferred, due to their busy schedules and fears of COVID-19:

I think the phone can be used for follow-up and support after getting one’s test results. In this era of COVID and with our busy schedules it’s very hard for me to keep going to the facility, but if I have somebody to continually offer guidance and information, I think it makes it easier. ([50], p. 8).

In the Zambian study, 31% (n = 203/647) of men preferred a phone call and 18% (n = 116/647) preferred SMS reminders [43]. One man reported:

I think both modes (phone and home visit) of talking to your counsellor are important, but if you want an effective communication, its better people come to your home so that you build trust and strong relationship ([43], p. 8).

In a study conducted in Malawi among men with partners attending antenatal care, follow up phone call reminders were preferred, and these increased linkage to HIV care among men who received HIVST kits from their partners [44, 45]. Phone call reminders after HIVST were also preferred over SMS reminders in Tanzania [46]. In a study conducted in South Africa, confirmatory testing and linkage to HIV care after a reactive HIVST result were low, despite telephone reminders and home visits, because some participants could not be reached at home or by telephone. Following up with SMS reminders after HIVST was not perceived as effective for improving linkage to HIV care, because some people simply ignored them due to an influx of spam messages [44]. Attempts by healthcare providers to provide repeated follow ups may deter future utilization of HIVST if these attempts are thought to compromise the autonomy of HIVST clients [52]. Some have also voiced concern that home visits could potentially stigmatize HIVST clients who are labelled as “HIV infected” [52].

A study conducted in KwaZulu-Natal, South Africa, indicated that men who received an HIVST kit at a place other than the workplace or mobile van (AOR 3.58; 95% CI 1.30–14.84; p = 0.033) and those with a secondary level of education or above (AOR 1.34; 95% CI 1.00–1.78; p = 0.046) were more likely to report their HIVST results [54]. In Rakai, Uganda, men suggested that peers could be used to encourage men with a reactive HIVST result to seek confirmatory results and, if confirmed to be HIV positive, link to HIV care as soon as possible:

A peer educator can intervene and take HIV treatment to the homes of the self-tested individuals… [The use of a peer-leader] could be effective because the peer leader can reach his colleague who self-tested HIV positive without informing others… it is better if they get linked through the peer leader who distributed the kits. ([49], p. 11).

## Discussion

In this systematic review of research on linkage to HIV care following HIVST in sub-Saharan Africa, we found a paucity of evidence overall. More research is required to understand barriers to confirmatory testing and ART linkage after a reactive HIVST result, as well as potential enabling factors, so that program implementers can design more effective linkage to care interventions aimed at improving the rates of men accessing HIV treatment in these settings [57].

Some studies described the potential of financial incentives to improve rates of linkage to confirmatory testing and subsequent HIV care following a reactive HIVST result among men. For those living in conditions of poverty, the financial incentives could be used for transport costs, removing a crucial economic barrier to ART linkage. However, it is important to note that this finding was based on only a few studies. More evidence is needed to confirm the impact of incentives on linkage to care, despite the promising results to date. As high as 72% of men obtained confirmatory testing and subsequently linked to HIV care following reactive HIVST results in South Africa [53], but inconsistent findings were noted in other settings [26, 43, 47, 55, 58]. In addition to financial barriers, other barriers include poor integration of HIV and primary healthcare services [43, 45, 47, 58], long wait times, stigma, discrimination and privacy concerns [8].

Given these persistent barriers deterring men from accessing HIV services in health care settings, our results suggest the importance of community-based confirmatory HIV testing and ART initiation. Community-based initiation and delivery of ART services has been shown to be an effective strategy for improving access to HIV treatment in sub-Saharan Africa, as it addresses several distinctive barriers associated with accessing HIV treatment from clinic settings [59–63].

The findings of this review make an important contribution to the literature on HIVST in sub-Saharan Africa, given the limited evidence on this important topic. Nevertheless, the findings should be interpreted in light of several important limitations. First, we did not include studies conducted in countries in sub-Saharan Africa that are not implementing the HIVST programme. Second, the findings of this review may not be generalised across the region, especially given the limited number of studies that met our inclusion criteria. Furthermore, most of the included studies were conducted in Eastern and Southern Africa, and few studies focused on secondary distribution of HIVST kits to the male partners of women attending antenatal care clinics. Third, we did not attempt to contact authors for gender-disaggregated data if they were not reported in the full text. Finally, in some included studies, it is possible that linkage to confirmatory testing and care was underestimated, especially in cases where participants could not be reached after follow-up attempts.

## Conclusion

HIVST is an important model for improving men’s uptake of HIV care and related services. The rates of men seeking confirmatory testing and linkage to HIV care following HIVST remain inconsistent. Measuring linkage to HIV care following a reactive HIVST result remain a primary concern in sub-Saharan Africa. This suggests that further research and strategies are required to address ongoing concerns over linkage to HIV care, especially given the contrasting findings.

## Data Availability

All the data analysed and reported in this paper were from published literature, which is already in the public domain.

## Appendix

See Table 3.

**Table 3:** Methodological quality assessment of included studies

Question number	Q1	Q2	Q3	Q4	Q5	Q6	Q7	Q8	Q9	Q10	Q11	Q12
Qualitative studies												
Muwanguzi et al. [50]	Y	Y	Y	Y	Y	Y	Y	N/A	N/A	N/A	N/A	N/A
Rujumba et al. [48]	Y	Y	Y	Y	Y	Y	Y	N/A	N/A	N/A	N/A	N/A
Matovu et al. [49]	Y	Y	Y	Y	Y	Y	Y	N/A	N/A	N/A	N/A	N/A
Conserve et al. [46]	Y	Y	Y	Y	Y	Y	Y	N/A	N/A	N/A	N/A	N/A
Choko et al. [44]	Y	Y	Y	Y	Y	Y	Y	N/A	N/A	N/A	N/A	N/A
Martinez Perez et al. [52]	Y	Y	Y	Y	Y	Y	Y	N/A	N/A	N/A	N/A	N/A
Makusha et al. [51]	Y	Y	Y	Y	Y	Y	Y	N/A	N/A	N/A	N/A	N/A
Quantitative studies												
Sithole et al. [54]	Y	Y	N/A	N/A	N/A	N/A	N/A	Y	Y	Y	Y	Y
Korte et al. [47]	Y	Y	N/A	N/A	N/A	N/A	N/A	Y	Y	Y	Y	Y
Choko et al. [45]	Y	Y	N/A	N/A	N/A	N/A	N/A	Y	Y	Y	C	Y
Masters et al. [55]	Y	Y	N/A	N/A	N/A	N/A	N/A	Y	Y	Y	Y	Y

Question number	Q1	Q2	Q13	Q14	Q15	Q16	Q17	Q18	Q19	Q20	Q21	Q22
Shapiro et al. [53]	Y	Y	Y	Y	Y	Y	Y	N/A	N/A	N/A	N/A	N/A
Thirumurthy et al. [56]	Y	Y	Y	Y	Y	Y	Y	N/A	N/A	N/A	N/A	N/A
Choko et al. [26]	Y	Y	Y	Y	Y	Y	Y	N/A	N/A	N/A	N/A	N/A
Mixed methods studies												
Chipungu et al. [43]	Y	Y	N/A	N/A	N/A	N/A	N/A	Y	Y	Y	Y	Y

Screening questions (for all types): Q1: Are there clear research questions?; Q2: Do the collected data allow to address the research questions?

Qualitative: Q3: Is the qualitative approach appropriate to answer the research question?; Q4: Are the qualitative data collection methods adequate to address the research question?; Q5: Are the findings adequately derived from the data?; Q6: Is the interpretation of results sufficiently substantiated by data?; Q7: Is there coherence between qualitative data sources, collection, analysis and interpretation?

Quantitative randomized controlled trials: Q8: Is randomization appropriately performed?; Q9: Are the groups comparable at baseline?; Q10: Are there complete outcome data?; Q11: Are outcome assessors blinded to the intervention provided?; Q12: Did the participants adhere to the assigned intervention?

Quantitative: Q13: Is the sampling strategy relevant to address the research question?; Q14: Is the sample representative of the target population?; Q15: Are the measurements appropriate?; Q16: Is the risk of nonresponse bias low?; Q17: Is the statistical analysis appropriate to answer the research question?

Mixed methods: Q18: Is there an adequate rationale for using a mixed methods design to address the research question?; Q19: Are the different components of the study effectively integrated to answer the research question?; Q20: Are the outputs of the integration of qualitative and quantitative components adequately interpreted?; Q21: Are divergences and inconsistencies between quantitative and qualitative results adequately addressed?; Q22: Do the different components of the study adhere to the quality criteria of each tradition of the methods involved?

*Y* yes; *N* no; *C* can’t tell; *N/A* not applicable

## Abbreviations

HIVST: HIV self-testing
MeSH: Medical Subject Headings
MMAT: Mixed Method Appraisal Tool
PCC: Population, Concept, and Context
PRISMA: Preferred Reporting Items for Systematic Reviews and Meta-Analysis
UCTD: Union Catalogue of Theses and Dissertations
UNAIDS: The Joint United Nations Programme on HIV and AIDS
WHO: World Health Organization

## Declarations

### Research Involving Human and/or Animal Participants

This review does not include any animal or human participants.
